# Notch regulates BMP responsiveness and lateral branching in vessel networks via SMAD6

**DOI:** 10.1038/ncomms13247

**Published:** 2016-11-11

**Authors:** Kevin P. Mouillesseaux, David S. Wiley, Lauren M. Saunders, Lyndsay A. Wylie, Erich J. Kushner, Diana C. Chong, Kathryn M. Citrin, Andrew T. Barber, Youngsook Park, Jun-Dae Kim, Leigh Ann Samsa, Jongmin Kim, Jiandong Liu, Suk-Won Jin, Victoria L. Bautch

**Affiliations:** 1 Department of Biology, The University of North Carolina at Chapel Hill, Chapel Hill, North Carolina 27599, USA; 2 Lineberger Comprehensive Cancer Center, The University of North Carolina at Chapel Hill, Chapel Hill, North Carolina 27599, USA; 3 Curriculum in Genetics and Molecular Biology, The University of North Carolina at Chapel Hill, Chapel Hill, North Carolina 27599, USA; 4 Department of Life Systems, Sookmyung Women's University, Seoul, Korea; 5 Section of Cardiovascular Medicine, Department of Internal Medicine, Yale Cardiovascular Research Center, School of Medicine, Yale University, New Haven, Connecricut 06511, USA; 6 Department of Pathology and Laboratory Animal Medicine, The University of North Carolina at Chapel Hill, Chapel Hill, North Carolina 27599, USA; 7 McAllister Heart Institute, The University of North Carolina at Chapel Hill, Chapel Hill, North Carolina 27599, USA; 8 School of Life Sciences and Cell Logistics Research Center, Gwangju Institute of Science and Technology, Gwangju, Korea

## Abstract

Functional blood vessel growth depends on generation of distinct but coordinated responses from endothelial cells. Bone morphogenetic proteins (BMP), part of the TGFβ superfamily, bind receptors to induce phosphorylation and nuclear translocation of SMAD transcription factors (R-SMAD1/5/8) and regulate vessel growth. However, SMAD1/5/8 signalling results in both pro- and anti-angiogenic outputs, highlighting a poor understanding of the complexities of BMP signalling in the vasculature. Here we show that BMP6 and BMP2 ligands are pro-angiogenic *in vitro* and *in vivo*, and that lateral vessel branching requires threshold levels of R-SMAD phosphorylation. Endothelial cell responsiveness to these pro-angiogenic BMP ligands is regulated by Notch status and Notch sets responsiveness by regulating a cell-intrinsic BMP inhibitor, SMAD6, which affects BMP responses upstream of target gene expression. Thus, we reveal a paradigm for Notch-dependent regulation of angiogenesis: Notch regulates SMAD6 expression to affect BMP responsiveness of endothelial cells and new vessel branch formation.

## Abstract (web summary)

The mechanism underlying endothelial cell responses to BMP signals is unknown. Here, the authors show that the endothelial response to pro-angiogenic BMP ligands is regulated by Notch via its effect on SMAD6, a known inhibitor of BMP intracellular signaling cascade.

## Graphical abstract

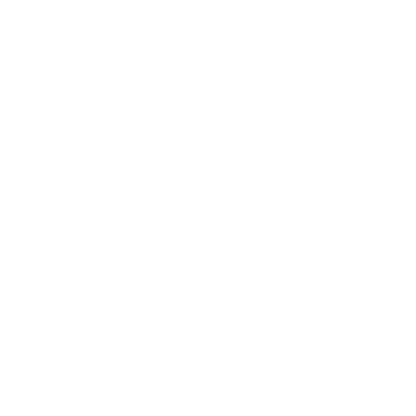

Blood vessel networks expand via endothelial cell (EC) sprouting, migration, anastomosis and lumenization to form new conduits, a process called sprouting angiogenesis^1^. Heterogeneous yet coordinated responses to pro-angiogenic signals are essential for proper angiogenesis and for subsequent maintenance of a functional vasculature^2^,^3^,^4^,^5^,^6^. Numerous signalling pathways integrate to provide this heterogeneity in ways that are not completely understood^5^,^7^,^8^.

Bone morphogenetic protein (BMP) signalling is essential to the proper form and function of blood vessels^8^,^9^,^10^,^11^,^12^,^13^,^14^. BMPs belong to the tranforming growth factor-β superfamily of secreted growth factors and they signal through cell-surface heterotetramers of type II and type I serine/threonine kinase receptors^15^,^16^. Phosphorylation of type I receptors by type II receptors induces phosphorylation of the receptor-associated SMADs 1, 5 and 8 (R-SMADs 1/5/8). R-SMAD phosphorylation induces association with SMAD4 and this complex translocates to the nucleus, to regulate expression of target genes. SMAD6 is an inhibitory SMAD that competitively binds type I receptors or SMAD4 to inhibit SMAD1/5/8 phosphorylation or nuclear translocation, respectively, and downregulate BMP signalling^17^,^18^.

Recent studies highlight novel roles for BMPs during angiogenesis^8^,^9^,^10^,^11^,^12^,^13^,^14^. Global genetic deletion of SMAD5 in mice led to multiple angiogenesis defects during embryogenesis^19^. EC-selective deletion of SMAD1 and SMAD5 severely impaired angiogenesis, resulting in defective yolk sac and cranial vasculature, and SMAD1/5 double knockout EC displayed reduced proliferation and migration, implicating BMP signalling in cellular processes essential to angiogenesis^8^. In zebrafish, Bmp2b is a venous-specific pro-angiogenic signal that promotes Vegfaa-independent sprouting from the posterior cardinal vein^9^. The type I BMP receptor ALK1 is either anti-angiogenic or pro-angiogenic when inhibited by Fc-conjugation or a highly specific blocking antibody, respectively^10^,^13^. These data suggest that the angiogenic activity of BMP ligands is context dependent.

EC differentially respond to BMP signalling^9^, but how BMP responsiveness is set remains largely unknown. Therefore, we sought to identify a mechanism by which EC intrinsically regulate the magnitude of their responses to BMP ligands. Previous studies indicated that BMP6 and BMP2 can be pro-angiogenic^8^,^9^,^20^. Here we show that these ligands are pro-angiogenic, and that lateral vessel branching requires threshold levels of R-SMAD phosphorylation. EC responsiveness to pro-angiogenic BMP ligands is regulated by Notch status. Notch sets responsiveness by regulating the cell-intrinsic BMP inhibitor, SMAD6 and SMAD6 affects BMP responses upstream of target gene expression, revealing a new paradigm for Notch-dependent regulation of angiogenesis.

## Results

### BMP2 and BMP6 promote lateral vessel branching

**Figure 1:**
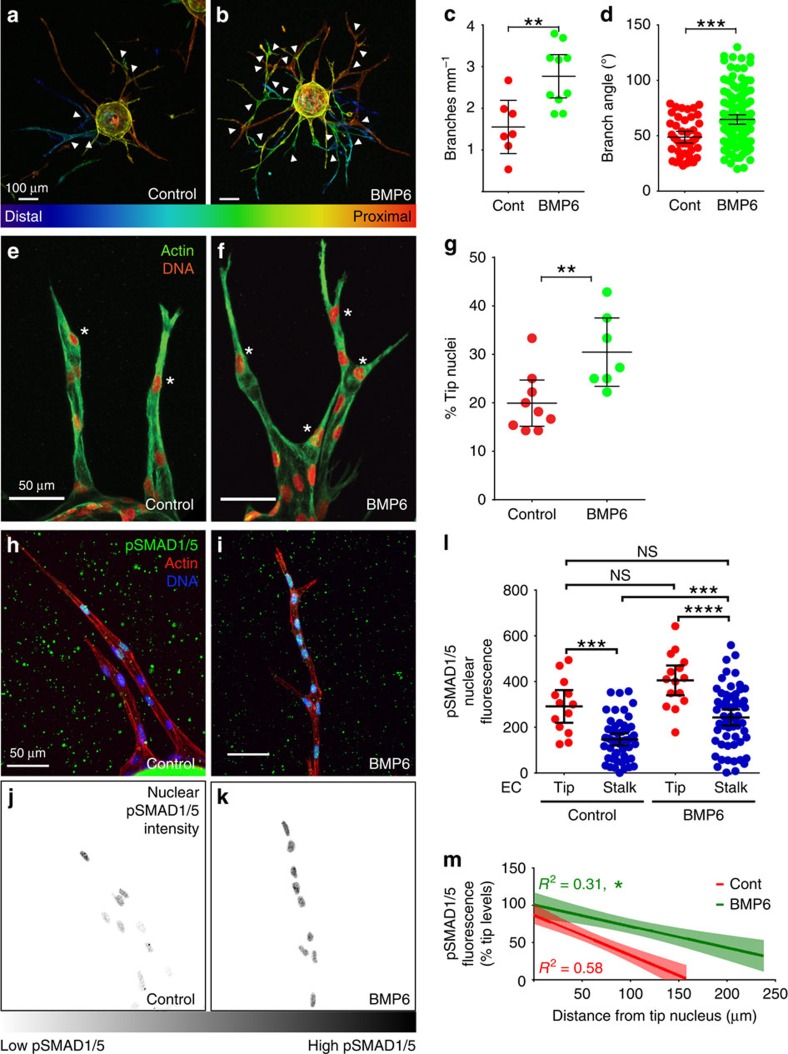
BMP signalling promotes lateral branching of angiogenic sprouts. (**a**,**b**) HUVEC 3D sprouting assay with BMP6, representative of three independent experiments, stained with phalloidin (actin) and depth encoded. (**c**,**d**) Quantification of (**c**) branches per mm (*n*=7 control and 10 BMP6 beads) and (**d**) branch angle (*n*=43 control and 133 BMP6 angles). Error bars, mean±95% confidence interval (CI). **P≤0.01; ***P≤0.001 by Student's *t*-test. (**e**,**f**) Sprouting HUVEC visualized with phalloidin (actin) and DRAQ7 (DNA). (**g**) Tip nuclei/total nuclei per field, representative of three independent experiments (*n*=9 control and 7 BMP6 sprouts). Error bars, mean±95% CI. **P≤0.01 by Student's *t*-test. (**h**–**k**) HUVEC 3D sprouting assay 6 h post-BMP6 treatment, visualized for nuclear pSMAD1/5, phalloidin (actin) and DRAQ7 (DNA). Images are three-channel compressed *z*-stacks (**h**,**i**) or single-channel compressed *z*-stack heat maps of pSMAD1/5 staining intensity (**j**,**k**). Scale bar, 50 mm. (**l**) Quantification of mean pSMAD1/5 fluorescence intensity per nucleus, representative of 2 independent experiments (*n*=13 control tip, 50 control stalk, 15 BMP6 tip, and 58 BMP6 stalk EC). Error bars, mean±95% CI. NS, not significant; ***P≤0.001 and ****P≤0.0001 by one-way analysis of variance with Tukey's *post-hoc* test. (**m**) Best-fit correlation (solid line) with 95% CI intervals (filled areas) of indicated parameters. *P≤0.05 by linear regression.

We first determined the effects of BMP6 and BMP2 on vessel branching in a three-dimensional (3D) sprouting angiogenesis assay^21^ using human umbilical vein endothelial cells (HUVEC). Addition of exogenous BMP6 or BMP2 significantly increased both branching frequency and branch angle compared with controls (Fig. 1a–d and Supplementary Fig. 1a–d). Exogenous BMP2 had a similar effect on branching frequency in mouse embryonic stem (ES) cell-derived vessels (Supplementary Fig. 1e–g). Consistent with a more highly branched phenotype, both ligands increased the percentage of nuclei in the tip position of growing HUVEC sprouts (Fig. 1e–g and Supplementary Fig. 1h–j). Moreover, the BMP-induced branching phenotype was remarkably well- organized, in contrast to the effects of elevated vascular endothelial growth factor (VEGF)-A signalling, which often lead to gross dysmorphogenesis^22^,^23^. Thus, BMP6 and BMP2 are pro-angiogenic and increase vascular density by inducing new branch formation. Furthermore, the increased branch angles lead to lateralization of the branching pattern and suggest that pro-angiogenic BMP signalling tunes vessel branching between a ‘bush-like' and a ‘bamboo-like' vessel pattern, in a ligand-dependent manner.

We hypothesized that BMP-induced branching depended on EC sprouting in response to activation and nuclear localization of phosphorylated SMADs 1 and 5 (pSMAD1/5). We examined levels of nuclear pSMAD1/5 in EC during sprouting angiogenesis and found a striking inverse correlation between the distance of nuclei from the sprout tip and intensity of nuclear pSMAD1/5 staining (Fig. 1h,j,m). Thus, EC with the highest levels of BMP signalling were more likely to be in the tip cell position. As expected, short-term BMP6 treatment significantly increased overall nuclear pSMAD1/5 levels compared with controls (Fig. 1i–k and Supplementary Fig. 1k). However, although acute BMP6 exposure significantly increased the average nuclear pSMAD1/5 levels in stalk cells, it had only a modest and nonsignificant effect in tip cells (Fig. 1l), suggesting that EC at sprout tips have maximal BMP pathway activation. In fact, stalk EC in BMP6-treated sprouts had average nuclear pSMAD1/5 levels comparable to levels in tip EC of controls (Fig. 1l), suggesting that BMP signalling increases vessel branching by inducing additional tip cells from the pool of stalk cells. This idea is also consistent with the finding that stalk cells, even after BMP stimulation, retain extensive heterogeneity in nuclear pSMAD1/5 levels and their pSMAD1/5 levels do not as strongly correlate with position in the sprout as controls (Fig. 1l,m).

To verify the necessity of SMAD1/5 for lateral branch formation, we reduced the levels of each protein via small interference RNAs (siRNAs) and analysed effects on sprouting. Reduction of either SMAD1 or SMAD5 (Supplementary Figs 1l,m and 5b) reduced BMP6-induced lateral branching. Although SMAD1/5 signalling has also been implicated in stalk cell maintenance^5^, this effect is thought to be downstream of BMP9 engagement with complexes that include the type I receptor ALK1 and induce vascular quiescence^13^,^24^,^25^. To determine whether ALK1 signalling contributes to the effects we observed, we reduced ALK1 levels via siRNA and found no effect on BMP6-dependent increases in lateral branching (Supplementary Figs 1n and 5b), suggesting that BMP6-induced branching occurs independently of BMP9/ALK1 signalling. Thus, pSMAD1/5 is required to mediate the pro-angiogenic effects of BMP6 in EC independent of effects from ALK1 signalling. Taken together, these data suggest that BMP-dependent lateral vessel branching depends on EC that are ‘tunable' and have an innate set point of BMP responsiveness. For each EC, this innate responsiveness sets a ligand threshold required to produce sufficient pro-angiogenic pSMAD1/5 signalling and provoke an EC response. In this scenario, short-term BMP6 treatment increases the number of EC that achieve the threshold and are capable of responding, leading to additional lateral branching.

### Notch sets EC BMP responsiveness

**Figure 2:**
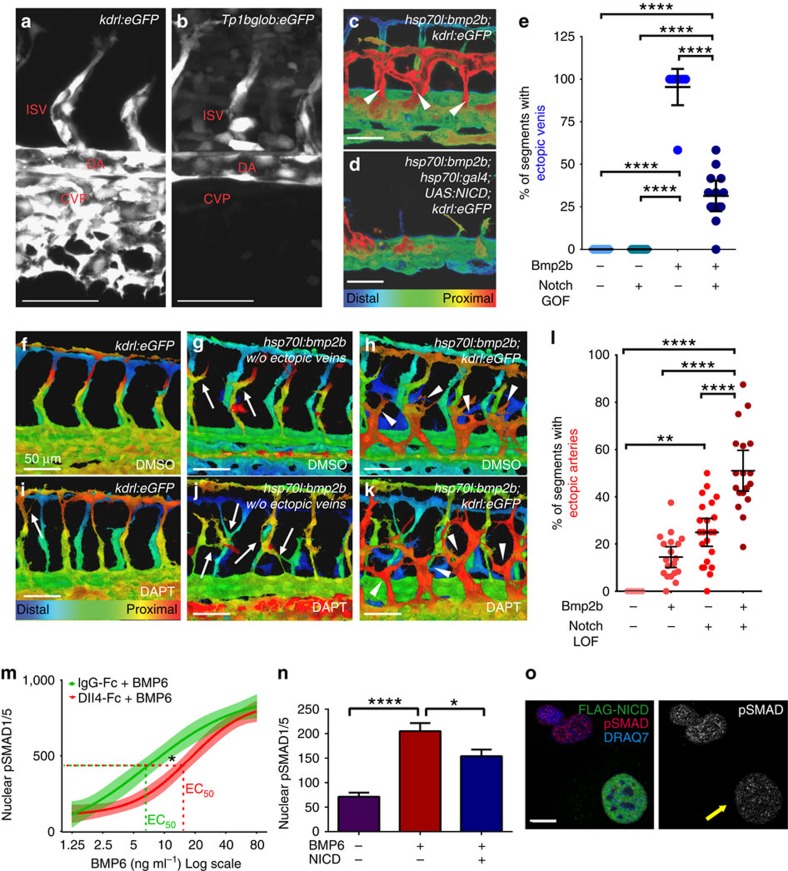
Notch regulates BMP responsiveness. (**a**,**b**) Lateral view of vessel-specific (**a**) or Notch-activated (**b**) GFP expression in 28 hpf fish embryos. Scale bar, 50 μm. (**c**,**d**) Depth-encoded lateral view of44 hpf fish embryos heat-shocked at 26 hpf. Scale bar, 50 μm. (**c**) *Tg*(*hsp70l:bmp2b)* embryos overexpress Bmp2b; (**d**) *Tg(hsp70l:bmp2b)*;*Tg*(*UAS:NICD); Tg(hsp70l:gal4)* overexpress both Bmp2b and NICD. (**e**) Quantification of ectopic venous sprouts, representative of two independent experiments. Data points, individual embryos (*n*=12 WT, 11 *Tg(hsp70l:Gal4);Tg(UAS:NICD)*, 9 *Tg(hsp70l:bmp2b)*, 13 compound transgenics). Error bars, mean±95% CI. ****P≤0.0001 by one-way analysis of variance (ANOVA), with Tukey's *post-hoc* test. (**f**–**k**) Depth-encoded compressed *z*-stack lateral views of 44 hpf embryos heat shocked at 26 hpf and treated with vehicle (**f**–**h**) or DAPT (**i**–**k**) from 10 hpf, representative of three independent experiments. (**f**,**i**) WT embryos; (**g**,**j**) *Tg*(*hsp70l:bmp2b)* embryos with ectopic venous *z*-slices removed to visualize intersegmental arteries; (**h**,**k**) *Tg*(*hsp70l:bmp2b)* embryos. (**l**) quantification of ectopic arterial sprouts. Data points, individual embryos (*n*=27 WT/dimethyl sulfoxide (DMSO), 26 Bmp2b/DMSO, 23 WT/DAPT and 26 Bmp2b/DAPT). Error bars, mean±95% CI. *P≤0.05, **P≤0.01 and ****P≤0.0001 by Kruskal–Wallis with Dunn's *post-hoc* test. DA, dorsal aorta; DV, dorsal vein; ISV, intersegmental vessel; VV, ventral vein. (**m**) BMP6 twofold dose–response curve (indicated on *x* axis) in HUVEC after Notch activation (Dll4-Fc, red line) versus control (IgG-Fc, green line), representative of two independent experiments. Data are four-parameter best-fit curves (solid lines) ±95% confidence bands (filled areas). *P≤0.05 by nonlinear regression. Quantification (**n**) and panels (**o**) of nuclear pSMAD1/5 expression in individual HUVEC with indicated conditions, representative of two independent experiments. Yellow arrow, EC expressing FLAG-NICD. Scale bar, 10 μm. Error bars, mean±s.e.m. *P≤0.05 and ****P=0.0001 by one-way ANOVA with Tukey's *post-hoc* test.

If BMP responsiveness is innate to EC, then factors that set this responsiveness and the extent of R-SMAD phosphorylation are predicted to profoundly affect BMP-dependent vascular architecture. Notch signals via its intracellular domain (NICD) and this signalling represses the tip cell phenotype and supports a non-branching stalk cell phenotype^26^,^27^,^28^,^29^. Although recent work describes Notch-BMP pathway crosstalk at the level of target gene expression^8^,^13^,^30^,^31^, it is not clear whether Notch signalling alters the responsiveness of EC to BMP via effects on upstream BMP pathway components. In the developing zebrafish, venous EC are BMP sensitive and form ectopic sprouts after heat-shock induction of Bmp2b, whereas intersegmental vessels (ISVs) are relatively unresponsive at 24–48 hpf (ref. 9). To investigate regulators of BMP responsiveness in this model, we used *Tg(Tp1bglob:eGFP)* (Notch reporter) zebrafish embryos and asked how Notch activity and BMP sensitivity align in the vasculature. Notch reporter activity was strong in the dorsal aorta (DA) and ISVs, consistent with other reports^32^,^33^, and these vessels do not respond to ectopic BMP ligand^9^; however, the reporter signal was undetectable in the BMP-responsive caudal vein plexus (CVP) (Fig. 2a,b). To determine whether Notch influences BMP responsiveness, we induced Notch signalling via heat-shock induction of NICD. As we have described, ectopic induction of Bmp2b led to excessive sprouting from the CVP (Fig. 2c,e)^9^. However, concomitant induction of Bmp2b and NICD significantly reduced the frequency of CVP sprouts, suggesting that ectopic Notch signalling dampens the sensitivity of EC to BMPs (Fig. 2d,e). Conversely, to determine whether arterial EC could be sensitized to Bmp2b overexpression, we blocked Notch signalling by treatment with N-[2S-(3,5-difluorophenyl)acetyl]-L-alanyl-2-phenyl-1,1-dimethylethyl ester-glycine (DAPT), a γ-secretase inhibitor that prevents cleavage and release of NICD. Bmp2b induction induced a low level of ectopic vessels from arterial EC (Fig. 2f–h,l) and DAPT treatment alone induced some ectopic arterial angiogenesis, consistent with previous reports (Fig. 2i,l)^5^,^27^. However, Notch inhibition combined with Bmp2b induction resulted in a significantly higher frequency of ectopic arteries compared with either manipulation alone (Fig. 2j–l). These results indicate that Notch is an intrinsic regulator of the magnitude of the BMP response in EC *in vivo*.

To quantitatively determine the impact of Notch manipulations on BMP pathway activation, we determined nuclear pSMAD1/5 levels on exposure of HUVEC to different amounts of ligand. A twofold serial dose–response curve to BMP6 yielded a prototypical sigmoidal semi-log curve for BMP-mediated EC activation, as measured by nuclear pSMAD1/5 levels (Supplementary Fig. 2a,b). We next tested the effect of Notch activation by plating HUVEC onto Fc-conjugated Dll4 ligand (Dll4-Fc) before short-term treatment with BMP6 and found that the EC_50_ for BMP-mediated EC activation increased significantly compared with controls (Fig. 2m). We confirmed, using inducible NICD expression in HUVEC, that elevated Notch signalling increased the EC_50_ (Supplementary Fig. 2c). This relationship also held at the single-cell level, as EC expressing NICD had reduced levels of pSMAD1/5 (Fig. 2n,o). Conversely, HUVEC treated with siRNA targeting Notch1 (Supplementary Figs 2d and 5c) were more sensitive to lower concentrations of BMP6 relative to controls (Supplementary Fig. 2e) and they exhibited increased branching with equivalent BMP6 stimulation (Supplementary Fig. 2f–j). The results of *in vivo* and *in vitro* Notch manipulations support our hypothesis that Notch regulates the innate BMP responsiveness of EC, and that the increased BMP responsiveness of EC with low Notch signalling promotes lateral branching.

### SMAD6 integrates Notch and pro-angiogenic BMP responsiveness

**Figure 3:**
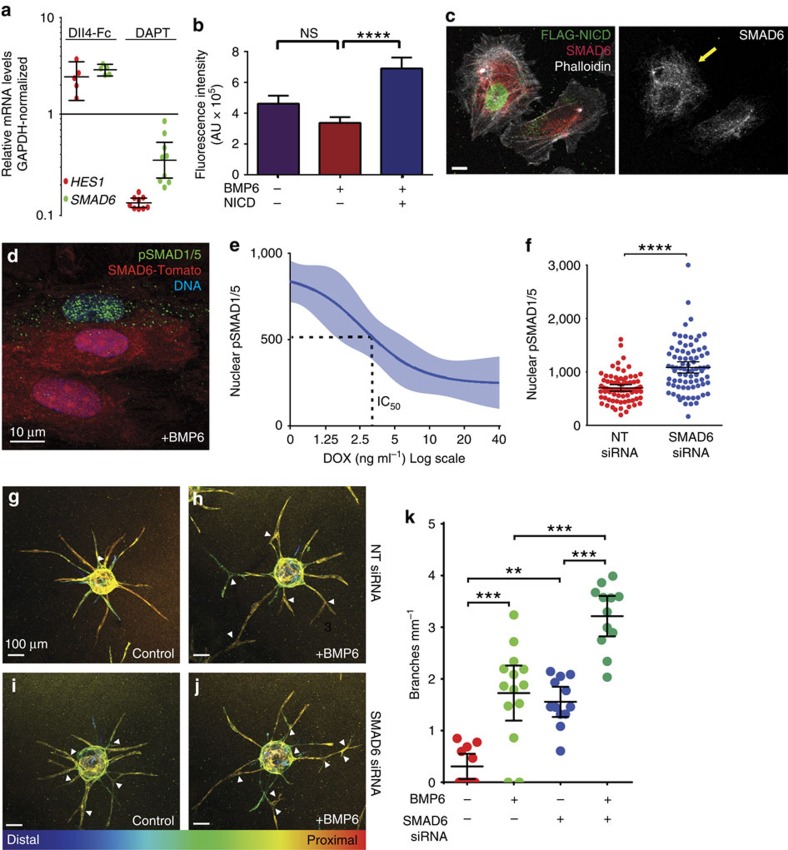
SMAD6 is Notch regulated and anti-angiogenic. (**a**) qRT–PCR for *HES1* or *SMAD6* with Notch gain-of-function (Dll4-Fc) or loss-of-function (DAPT). Data points, independent experiments (*n*=5 DLL4-Fc and 9 DAPT), log-conversion of the DDC_T_ versus controls. Quantification (**b**) and panels (**c**) of nuclear SMAD6 expression in individual HUVEC with indicated parameters, representative of two independent experiments. Scale bar, 10 μm. Yellow arrow, EC expressing FLAG-NICD. Error bars, mean±s.e.m., ****P<0.0001 by one-way analysis of variance using Tukey's *post-hoc*; NS, not significant. (**d**) Stable lentivirally transduced HUVEC infected with SMAD6-tdTomato conditonally expressing virus, stimulated with doxycycline (DOX) and stained as indicated. (**e**) Quantification of twofold dose–response (indicated on *x* axis) to DOX (SMAD6-tdTomato expression) after 90 min BMP6 treatment, representative of two independent experiments. Readout is nuclear pSMAD1/5. Data are four-parameter best-fit curves (solid lines) ±95% confidence bands (filled areas). (**f**) HUVEC transfected with indicated siRNAs, then treated with 40 ng ml^−1^ BMP6 for 90 min before fixation and staining for nuclear pSMAD1/5, representative of two independent experiments (*n*=75 NT and 80 SMAD6 siRNA HUVEC). Error bars, mean±95% CI. ****P≤0.0001 by Student's *t*-test. (**g**–**j**) HUVEC 3D sprouting assay with indicated siRNAs and treatment, visualized with phalloidin (actin) and depth-encoded in a compressed *z*-stack, representative of three independent experiments. (**k**) Quantification of branching (*n*=11 NT/control, 13 NT/BMP6, 12 SMAD6 siRNA/control and 11 SMAD6 siRNA/BMP6 beads). Data points, individual beads; bars, mean±95% CI. **P≤0.01 and ***P≤0.001 by Kruskal–Wallis with Dunn's *post-hoc* test.

Notch regulates VEGF signalling by modulating levels of VEGF receptor RNAs^34^,^35^. Therefore, we reasoned that Notch would regulate BMP responsiveness via expression of BMP receptors. Surprisingly, we detected no significant changes in expression levels of several type I and type II BMP receptors after Notch stimulation of HUVEC via Dll4-Fc plating or Notch blockade via DAPT (Supplementary Fig. 3a,b). BMP signalling is also intrinsically regulated by an intracellular inhibitory protein, SMAD6 (refs 17, 18, 36, 37) and SMAD6 messenger RNA levels increased with Notch stimulation and decreased with Notch blockade in HUVEC (Fig. 3a). This relationship was maintained at the single-cell level, as EC expressing NICD had elevated levels of SMAD6 protein (Fig. 3b,c). In other cell types, SMAD6 inhibits BMP signalling by preventing R-SMAD phosphorylation and nuclear localization^17^,^18^, but its activity in EC and effects on angiogenesis are unknown. Therefore, we generated HUVEC expressing doxycycline-inducible SMAD6 fused to tdTomato. The tagged SMAD6 protein reacted with a SMAD6 antibody by immunofluorescence (Supplementary Fig. 3c–f) and suppressed nuclear pSMAD1/5 levels in HUVEC in a cell autonomous and dose-dependent manner (Fig. 3d,e). Conversely, reduction of SMAD6 protein levels via siRNA knockdown (Supplementary Figs 3g and 5d) increased BMP6-induced nuclear pSMAD1/5 (Fig. 3f). These findings show that an intrinsic BMP pathway inhibitor, SMAD6, modulates BMP signalling in EC. As elevated pSMAD levels were associated with increased lateral branching and SMAD6 suppressed BMP signalling, we hypothesized that loss of SMAD6 would increase BMP responsiveness of EC and promote branching. Consistent with this hypothesis, reduced SMAD6 levels via siRNA significantly increased branching of sprouting HUVEC with added exogenous BMP6 (Fig. 3g–k). These results show that SMAD6 regulates angiogenesis, probably by intrinsically modulating the magnitude of EC responses to BMP inputs.

**Figure 4:**
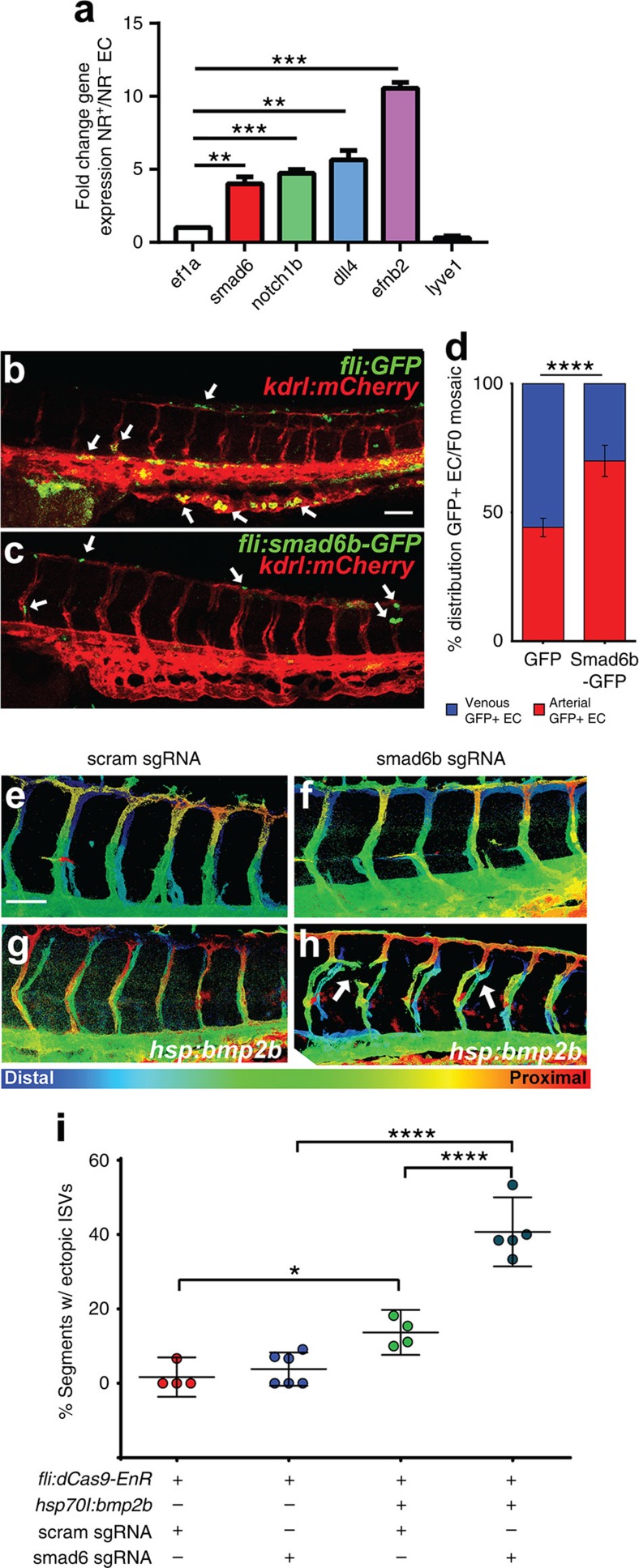
SMAD6 regulates BMP-dependent angiogenesis *in vivo.* (**a**) Quantitative RT–PCR for indicated genes, fold-change Notch reporter+ (NR+)/Notch reporter- (NR-) EC, relative to e1fa. Error bars, mean±s.e.m., *N*=5 replicates; one-sample Student's *T*-test, **P≤0.01 and ***P≤0.001. (**b**–**d**) F0 mosaic transgenic zebrafish embryos (44 hpf) expressing GFP control (**b**) or GFP-tagged smad6b (**c**) from the vessel-specific *fli* promoter in the *Tg(kdrl:mCherry)* line. Arrows, GFP+ EC. (**d**) Arterial versus venous distribution of GFP+ EC quantified as percentage of total GFP+ EC on a per-embryo basis. Data bars, average per cent arterial and venous GFP+ EC, ±95% CI, representative of three independent experiments (*n*=18 fli:GFP and 30 fli:smad6b-GFP F0 embryos). ****P≤0.0001 by *χ*^2^ analysis (1 degree of freedom). (**e**–**h**) Heat-shocked F1 embryos (heat shock at 26 hpf, analysed at 44–46 hpf) from *Tg(fli:dCas9-EnR)* and *Tg(hsp70l:bmp2b;Tg(kdrl:GFP)* crosses, injected with scrambled (scram) or with *smad6b* sgRNAs. Arrows, ectopic ISV sprouts. (**g**,**h**) have *Z*-planes with ectopic venous sprouts removed. (**i**) Quantification of arterial vascular defects (% segments with ectopic ISVs) in heat-shocked embryos of indicated genotypes, representative of two independent experiments (scram, *n*=4; smad6, *n*=6; scram/hsbmp, *n*=4; smad6/hsbmp, *n*=5). Error bars, mean±s.e.m.; *P≤0.05 and ****P≤0.0001 by one-way analysis of variance, with Tukey's *post-hoc* test.

To determine the functional relevance of SMAD6 *in vivo*, we first examined expression by fluorescence-activated cell sorting (FACS) in EC from zebrafish embryos that expressed the Notch signalling reporter (Fig. 2a). Zebrafish have two SMAD6 orthologues, *smad6a* and *smad6b*. We assessed expression of *smad6b* in EC *in vivo*, because its sequence is more homologous to that of human *SMAD6*. EC sorted for high levels of Notch reporter signalling had significantly elevated levels of RNAs that are expected to be elevated in arterial Notch-positive EC such as *notch1b* and *ephrinb2*. They also had significantly elevated levels of *smad6b* RNA relative to EC sorted from the same embryos with little or no Notch reporter signalling (Fig. 4a). We next manipulated embryonic SMAD6 expression. As global manipulations of *smad6b* are predicted to profoundly perturb dorsal–ventral axis formation in zebrafish embryos^38^, we used the Tol2 system to generate F0 mosaic embryos expressing green fluorescent protein (GFP)-tagged *smad6b*, or GFP alone, under control of the vascular-specific *fli1* promoter. We predicted that Smad6b expression would preferentially affect BMP-responsive EC with low Notch levels, such as those in the cardinal vein and the CVP. EC expressing Smad6b-eGFP were significantly reduced in the cardinal vein and CVP, and enriched in the DA and ISVs, compared with EC expressing only GFP (Fig. 4b–d), consistent with our finding that Smad6b expression is elevated in EC of embryos with high Notch signalling reporter expression, presumably including EC from the DA and ISVs. These findings suggest that forced Smad6b expression prevents EC from colonizing the vein and CVP where Notch signalling is low, but is irrelevant in arterial EC where Notch signalling is relatively high and support a role for SMAD6 in angiogenesis *in vivo*.

We next asked whether reduced levels of *smad6b* affect blood vessel branching and predicted that if SMAD6 is important for Notch-mediated effects *in vivo*, its reduction would more profoundly affect Notch-dependent ISV sprouting that is normally refractory to BMP^9^. To overcome the effects of global *smad6b* manipulations on dorsal–ventral axis formation, we developed a tissue-specific mRNA knockdown system for zebrafish based on a CRISPRi strategy^39^,^40^,^41^,^42^. However, CRISPRi is poorly efficient in eukaryotic cells^40^; thus, dCas9 was fused to the *Drosophila* engrailed repressor domain^43^ to generate dCas9-Engrailed Repressor domain (EnR), a chimeric transcriptional repressor protein that is targeted by short guide (sg) RNAs. We validated the efficacy of dCas9-EnR in global knockdown using sgRNAs targeting *bmp6* or *smad6b* and observed the expected dorsalization (*bmp6* loss-of-function) or ventralization (*smad6b* loss-of-function) phenotypes (Supplementary Fig. 4a–d). We also validated sgRNA effects at the mRNA level by quantitative reverse transcriptase–PCR (qRT–PCR) for the targeted genes (Supplementary Fig. 4e). Next, transgenic embryos expressing a *fli1* promoter-driven *dCas9-EnR* were generated using the Tol2 system. F1 embryos expressing vascular restricted dCas9-EnR were indistinguishable from wild-type (WT) siblings and *dCas9-EnR* mRNA expression was confirmed by RT–PCR (Supplementary Figs 4f–h anf 5e). To determine whether Smad6b regulates BMP responsiveness *in vivo*, we injected sgRNAs targeting *smad6b* into *Tg(fli1:dCas9-EnR);Tg(hsp701:bmp2b);Tg(kdrl:GFP)* embryos, then heat shocked to induce ectopic Bmp2b expression at 26 hpf, well beyond the early developmental window leading to axis defects. WT heat-shocked embryos without sgRNAs had normal ISV formation (Supplementary Fig. 4i,j,m). Likewise, *Tg(fli:dCas9-EnR);Tg(kdrl:GFP)* embryos injected with scrambled sgRNAs or smad6b sgRNAs and heat-shocked had normal ISV formation (Fig. 4e,f,i). Heat-shock induction of Bmp2b expression did not perturb axis formation, but induced some ectopic ISV formation in controls and *Tg(fli:dCas9-EnR); Tg(hsp701:bmp2b);Tg(kdrl:GFP)* embryos injected with scrambled sgRNAs (Fig. 4g,i and Supplementary Fig. 4k,m). However, the frequency of ectopic ISV formation significantly increased in transgenic embryos overexpressing Bmp2b and injected with smad6b sgRNAs (Fig. 4h,i; Supplementary Fig. 4l,m). Remarkably, the phenotype of BMP-overexpressing, vascular *smad6b* knockdown embryos resembles that of BMP-overexpressing embryos with reduced Notch signalling. Combined, these *in vivo* results support a model whereby SMAD6 represses BMP responsiveness and vessel branching.

**Figure 5:**
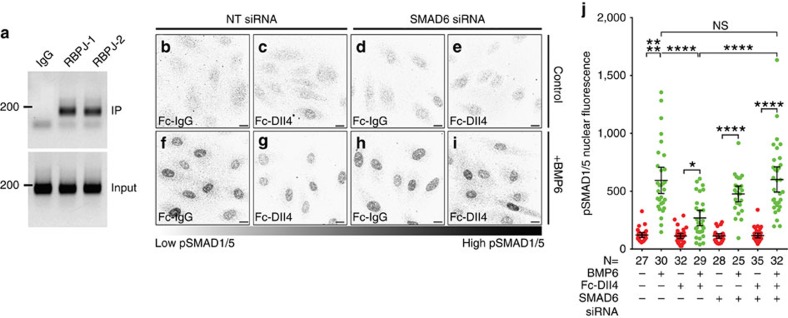
SMAD6 mediates Notch-dependent suppression of BMP signalling. (**a**) ChIP assay on micrococcal nuclease-digested HUVEC DNA immunoprecipitated with indicated antibodies and amplified unique primers targeting a single putative RBPJ consensus sequence in the SMAD6 promoter. Left, size marker (bp). (**b**–**i**) HUVEC transfected with non-targeting (**b**,**c**,**f**,**g**) or SMAD6 siRNA (**d**,**e**,**h**,**i**), plated onto IgG (**b**,**d**,**f**,**h**) or Dll4-Fc (**c**,**e**,**g**,**i**), then treated with control (**b**–**e**) or 50 ng ml^−1^ BMP6 (**f**–**i**) for 90 min before staining for nuclear pSMAD1/5. Scale bar, 10 μm. (**j**) Quantification of nuclear pSMAD1/5 fluorescence intensity, representative of three independent experiments. Data points, individual nuclei (n indicated on graph); bars, mean±95% CI. *P≤0.05, **P≤0.01 and ****P≤0.0001 by Kruskal–Wallis with Dunn's *post-hoc* test.

Based on the similarity of phenotypes *in vivo* and the Notch responsiveness of *SMAD6* RNA and protein in EC, we further explored the mechanism by which Notch regulates *SMAD6* gene expression. Analysis of the *SMAD6* 5′-promoter region revealed a consensus sequence for Recombination Signal Binding Protein for Immunoglobulin Kappa J Region (RBPJ) binding to DNA, which is required for Notch-dependent transcription in cells. A chromatin immunoprecipitation (ChIP) assay using two separate antibodies to RBPJ revealed that RBPJ binds at the identified site in the *SMAD6* promoter (Fig. 5a and Supplementary Fig. 5a), suggesting that Notch directly regulates transcription of *SMAD6* RNA.

Finally, we hypothesized that SMAD6 is functionally downstream of Notch in regulating the BMP responsiveness of EC. We tested this hypothesis by activating Notch in HUVEC with reduced levels of SMAD6, and we assessed BMP pathway signalling via nuclear localization of pSMAD1/5. Short-term BMP6 treatment of HUVEC led to elevated nuclear pSMAD levels that were significantly suppressed by Notch stimulation (Fig. 5b,c,f,g,j). In contrast, HUVEC with reduced SMAD6 levels and BMP6 stimulation did not respond to Notch stimulation with suppression of SMAD1/5 activation, but had nuclear pSMAD1/5 levels equivalent to that of HUVEC without Notch stimulation (Fig. 5d,e,h–j). These data indicate that SMAD6 is functionally downstream of Notch and is required to mediate the effects of Notch on BMP responsiveness in EC.

## Discussion

**Figure 6:**
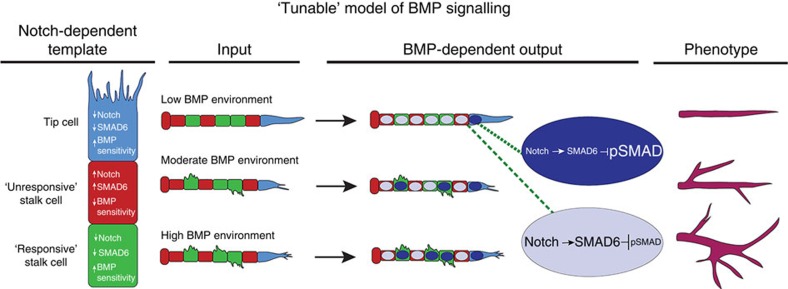
Model of SMAD6-mediated BMP responsiveness in vessels. The data support a model whereby Notch sets BMP responsiveness of sprouting EC through the intrinsic BMP inhibitor SMAD6, leading to a ‘tunable' system that responds to increased BMP ligands with more lateral branching.

Collectively, our data support a model whereby pro-angiogenic BMPs increase vascular complexity through regulation of lateral branching. The magnitude of intrinsic BMP responsiveness in EC is modulated by Notch-mediated regulation of the BMP inhibitor SMAD6 to ‘tune' BMP responsiveness (Fig. 6), and combined effects on branch frequency and angle lead to a more arborized or ‘bush-like' vascular network as BMP ligand levels increase. In this model, Notch signalling sets levels of SMAD6 in a given EC and SMAD6 levels regulate the amount of pSMAD1/5 that translocates to the nucleus with a given input of BMP ligand. As ligand levels increase, more EC reach a threshold that allows them to assume a tip cell phenotype and sprout to form a new branch. This includes a subset of EC in a classic ‘stalk' position, and our finding of differential BMP responsiveness in the stalk cell compartment indicates dissimilarity among stalk cells. Notch is a major regulator of the tip cell versus stalk cell phenotype in EC of angiogenic sprouts^26^,^44^, and we now propose that Notch also differentiates stalk cells by modulating expression of SMAD6. We predict that Notch-dependent changes in SMAD6 expression among dynamically competing sprouting EC shifts BMP responsiveness relative to a threshold necessary for lateral branching. Subsequently, as arteries reach homeostasis and Notch signalling becomes more uniform, these changes may lead to more uniform SMAD6 levels and overall dampened BMP responsiveness in arteries versus veins.

Allthough convergence of Notch and BMP pathways at downstream target genes has been described^8^,^13^,^30^, our data strongly suggest that intrinsic responsiveness to BMP signals is independently preset by Notch to regulate lateral branching. Recent work suggests that Notch also maintains the stalk cell phenotype via downregulation of Neuropilin-1 (ref. 45). Low Neuropilin-1 in stalk cells relieves inhibition of ALK1 and ALK5, and promotes tranforming growth factor-β-mediated pSMAD2/3 signalling to maintain the stalk cell phenotype. Our work shows that SMAD6, a Notch-regulated cell-intrinsic BMP pathway inhibitor, modulates EC responses to pro-angiogenic BMP ligands that stimulate pSMAD1/5 signalling and a tip cell phenotype. Thus, Notch-mediated SMAD6 regulation ‘tunes' branching responses to BMP ligands among stalk cells and coordinately regulates the magnitude of BMP pathway activation and sprouting, to determine the patterning of growing vessel networks.

## Methods

### Cell maintenance and processing

No cell lines used in this study are found in the International cell line authentication committee (ICLAC) commonly misidentified cell line database.

Mouse ES cells were maintained and differentiated as previously described^46^. Briefly, cells were maintained undifferentiated by culture in DMEM media supplemented with conditioned media from 5,637 human bladder cancer cells (ATCC HTB9) and passaged at 2–4 days. For differentiation, ES cells aged for 2–4 days beyond passage were trypsinized, resuspended and inoculated into droplets on a tissue culture plate lid. The lid was inverted, hanging drops cultured for 2 days to generate embryoid bodies (EBs), then inverted again and flushed with media. After culture in a new plate for 1 day, EBs were seeded at 20–30 EBs per well of a 24-well plate and differentiated in DMEM, Hi-glucose, 20% FCS, 150 μM monothioglycerol and 50 μg ml^−1^ gentamicin. Media was changed every 2 days until fixation. Human BMP2 (R&D Systems 355-BEC) was added at d6 and d7 at 200 ng ml^−1^. Day 8 cultures were rinsed with 1 × PBS and fixed for 5 min in ice-cold methanol-acetone (50:50)^47^. Following 6 × 5 min washes in 1 × PBS, cultures were stained with rat anti-mouse PECAM-1 (BD Biosciences 553370) at 1:1,000 and then goat anti-rat IgG Alexa Fluor 488 (Life Technologies A-11006) at 1:2,000 overnight at 4 °C in Staining Solution (Jackson ImmunoResearch 005-000-121), 5% goat serum, 1% BSA and 0.3% TritonX-100 in DPBS (Fisher MT-21-031-CV).

HUVEC (Lonza C2519A) were maintained as per the manufacturer's recommendations and were used in experiments from passages 2 to 5. All experiments were independently replicated using two distinct lots of HUVEC. HUVEC were certified mycoplasma-free by the UNC Tissue Culture Facility. Normal Human Lung Fibroblasts (NHLF; Lonza CC-2512) were maintained as per the manufacturer's recommendations. NHLF were certified mycoplasma-free by the UNC Tissue Culture Facility.

To induce Notch signalling, HUVEC were grown on recombinant protein G (Life Technologies 10–1201)-immobilized 10 μg ml^−1^ Fc-Dll4 (Adipogen AG-40A-0077-C050)^48^ (or 10 μg ml^−1^ Fc-IgG control (Life Technologies 10374-H02H-50)) for 24 h, with BMP6 treatments and immunostaining performed as described above. For doxycycline induction, stably infected HUVEC FLAG-NICD cells or controls (see below) were treated for 24 h with 1–2 ng ml^−1^ doxycycline (Sigma-Aldrich D9891-1G) in growth media, then incubated with BMP6 (200 ng ml^−1^) for 2 h, fixed in 4% paraformaldehyde (PFA) for 15 min, rinsed three times with PBS, permeabilized with 0.5% Triton-X for 20 min and then blocked overnight with 1% BSA/0.3% Triton-X/5% goat serum in PBS.

### Sprouting angiogenesis assay

The 3D sprouting angiogenesis assay was performed as previously described^21^. Briefly, 1–2 × 10^6^ HUVEC were mixed with 100 μl of a 60,000 ml^−1^ suspension of cytodex beads (GE Healthcare 17-0485-01) in 5 ml HUVEC growth media (EBM-2+EGM-2 BulletKit, Lonza CC-3156 and CC-3162, respectively), maintained in suspension for 4 h by mixing every 15 min, then placed in 60 mm dishes at 37 °C overnight. Beads were recovered from the plate by gentle rinsing and washed 3 × in HUVEC growth media, then resuspended in 10 ml of 2 mg ml^−1^ fibrinogen/0.15 U ml^−1^ aprotinin. Five microlitres of a 50 U ml^−1^ thrombin solution was added to each well of a 24-well plate, followed by gently mixing in 500 μl of HUVEC-coated beads in fibrinogen/aprotinin per well. After 5 min atRT, the plate was transferred to 37 °C for 30 min for polymerization, then 1 ml of 20,000 cells ml^−1^ NHLF was added to each well. Media was refreshed every 2 days until fixation. Recombinant human BMP2 or BMP6 (R&D Systems #355-BM-010 or #507-BP-020, respectively) were added at d2–6 at 200 ng ml^−1^. Day 7 cultures were fixed in 2% PFA (Electron Microscopy Supplies #15713) in DPBS (Fisher #MT-21-031-CV) for 30 m, then stained with Alexa Fluor 488 Phalloidin or Alexa Fluor 594 Phalloidin (Life Technologies #A12379 or #A12381, respectively) overnight at 4 °C at 1:100 in Staining Solution (5% Goat Serum (Jackson ImmunoResearch #005-000-121), 1% BSA, and 0.3% TritonX-100 in DPBS). Nuclei were counterstained with DRAQ7 (Abcam #ab109202) for 5 min at 1:1,000 in DPBS.

### Immunofluorescence

For pSMAD1/5 immunofluorescence, HUVEC were grown for 24 h on 0.1% gelatin (Sigma-Aldrich #G9136-10MG) coated 12 mm diameter #1.5 glass coverslips (Fisher #12-545-81). HUVEC were pretreated for 4 h in OptiMEM (Life Technologies #31985-070)+0.1% normal bovine calf serum (NBCS), then treated for 90 min with BMP6 at the doses described in each figure. Cells were fixed for 10 min in 4% PFA/DPBS, followed by permeabilization in 0.5% Triton-X 100/DPBS. Cells were blocked in staining solution for 1 h, followed by overnight incubation at 4 °C with Rabbit anti-pSMAD1/5 (R&D) at 1:1,000 in staining solution (see Supplementary Table 1 for antibodies and concentrations). Goat anti-Rabbit Alexa Fluor 488 secondary antibody was added at 1:1,000 in staining solution for 2 h at room temperature (RT). For FLAG experiments, HUVEC were incubated with 1:1,000 α-pSMAD 1/5 antibody (R&D) and 1:5,000 α-FLAG antibody (Sigma) overnight, then washed and incubated with 1:500 Alexa Fluor 594 to detect pSMAD 1/5 and with 1:5,000 Alexa Fluor 488 to detect FLAG. DRAQ7 (1:1,000) was used to visualize the nucleus. Coverslips were mounted onto slides in ProLong Diamond Antifade Mountant (Life Technologies P36961).

For SMAD6 staining, HUVEC were incubated in 1:100 α-SMAD6 (Abcam) and 1:5,000 α-FLAG antibody (Sigma) overnight, and then with 1:500 Alexa Fluor 594 to detect SMAD6 and with 1:5,000 Alexa Fluor 488 to detect the FLAG antibody. Alexa Fluor 647-conjugated Phalloidin was used to visualize cell boundaries.

### Plasmids

To generate a lentiviral vector expressing human SMAD6-tdTomato fusion protein under the control of a doxycycline-inducible promoter, the full-length human SMAD6 coding sequence was amplified from pOTB7 hSMAD6 (GE Dharmacon MHS6278–202829590) using PrimeSTAR MAX polymerase (Clontech R045A) and the following primers:

Forward: 5′-ATTCACAGATCTGCCACCATGTTCAGGTCCAAACGCTCG-3′, containing 6 nt overhang, BglII restriction site and Kozak consensus sequence,

Reverse: 5′-ATTCACGGTACCCTTCTGGGGTTGTTGAGGAGGATCTC-3′, containing 6 nt overhang, KpnI restriction site and 1 nt deletion from stop codon for in-frame read-through to carboxy-terminal tdTomato tag.

PCR product was purified using NucleoSpin kit (Clontech 740609) and double-digested for 10 min at 37° with BglII and KpnI (Life Technologies FD0084 and FD0524, respectively). ptdTomato-N1 empty vector (Clontech 632532) was double-digested in parallel using the same enzymes and de-phosphorylated for 5 min at 37° using FastAP Thermosensitive Alkaline Phosphatase (Life Technologies EF0654). Products were ligated at a 3:1 (Insert:Vector) molar ratio for 10 min at RT using T4 Rapid Ligation Kit (Life Technologies K1422), followed by transformation, selection, plasmid purification and sequencing, to verify the fidelity of the insert.

The full-length hSMAD6-tdTomato chimera was PCR-amplified and cloned into gateway-compatible pME-MCS^49^ from the newly generated template using a similar strategy.

Forward primer: 5′-ATTCACAAGCTTGCCACCATGTTCAGGTCCAAACGCTCG-3′, containing 6 nt overhang, HindIII (Life Technologies FD0504) restriction site and Kozak consensus sequence,

Reverse primer: 5′-ATTCACTCTAGACTACTTGTACAGCTCGTCCAT-3′, containing 6 nt overhang and XbaI (Life Technologies FD0684) restriction site.

An LR reaction was performed using Clonase II Plus (Life Technologies 12538-120), pME-hSAMD6-tdTomato and pLIX-402 EV (a gift from David Root (Addgene plasmid 41394)) to generate pLIX-402 hSMAD6-tdTomato.

pLIX-402 3 × -FLAG-mN1ICD was generated from pCMV-7 3 × -FLAG mN1ICD^50^ (a gift from Raphael Kopan, Addgene plasmid 20183) by double digestion and ligation into pME-MCS as described above, using FastDigest EcoRI and BamHI (Life Technologies FD0274 and FD0054, respectively), followed by an LR reaction.

Zebrafish transgenesis was performed using the Tol2 system^49^. To generate the pTol2 *fli1:smad6b-GFP;cmlc2:GFP* targeting vector, full-length *smad6b* was amplified from a 24 hpf embryo complementary DNA library using PrimeSTAR Max and cloned into pCS2 FLAG EV (a gift from Peter Klein, Addgene plasmid 16331) as described above, using the following primers:

Forward: 5′-ATTCACGGATCCGCCACCATGTTCAGGACGAAACGCTCA-3′, containing a 6 nt overhang, BamHI site and Kozak consensus sequence,

Reverse: 5′-ATTCACATCGATATCTGTGGTTGTTGAGGAGG-3′, containing a 6 nt overhang, ClaI (Life Technologies FD0143) site and 2 nt deletion from the stop codon to maintain frame with C-terminal tags.

C-terminal FLAG tag was replaced with GFP by amplifying GFP from peGFP-N1 EV (Clontech 6085-1), digested and ligated as described above, using the following primers:

Forward: 5′-ATTCACATCGATTGGTGAGCAAGGGCGAGGAGC-3′, containing a 6 nt overhang, ClaI site and a 1 nt deletion from the start codon,

Reverse: 5′-ATTCACAGGCCTTTACTTGTACAGCTCGTCCAT-3′, containing a 6 nt overhang and StuI (NEB R0187S) site.

*Smad6b-GFP* was then ligated into pME-MCS using a BamHI and NotI (Life Technologies FD0594) double digest as described above. A four-way LR reaction using p5E-*fli1ep* (a gift from Nathan Lawson, Addgene plasmid 31160), pME-*smad6b-GFP*, p3E-polyA^49^ and pDEST Tol2 CG2 (ref. 49) produced the final targeting vector.

To generate the pTol2-*fli1ep:Cas9-EnR*;*cmlc2:GFP* targeting vector, *dCas9* was amplified from pdCas9 (ref. 41; a gift from Stanley Qi, Addgene plasmid 44246) and ligated into pCS2-*EnR*^43^ (a gift from Ramesh Shivdasani, Addgene plasmid 11028) using the following primers:

Forward: 5′-GGATCCGCCACCATGGACAAGAAGTATTCTATC-3′, containing a 6 nt overhang, BamHI site and Kozak consensus sequence,

Reverse: 5′-ATTCACATCGATTACCAATGCCGTCTACCTT TCTCTTCTTTTTTGGATCTACCTTTCTCTTCTTTTTTGGATCTACCTT-3′, containing a 6 nt overhang, ClaI site and deletion of the stop codon.

*dCas9-EnR* was subcloned into pME-MCS using BamHI/XbaI double digests and four-way LR reaction performed with the same 5E, 3E and DEST vectors described above.

### Lentivirus production and stably infected HUVEC

Lenti-X 293T cells (Clontech 632180) were maintained in DMEM Hi Glucose (Life Technologies 11995-065)+10% normal bovine calf serum (NBCS) according to the manufacturer's instructions. For lentiviral production, cells were plated onto 10 cm dishes at 50,000 cm^−2^. The following morning, cells were co-transfected with 20 μg pLIX 402 vectors (described above) and 6 μg pMD2.G (a gift from Didier Trono, Addgene plasmid 12259), 5 μg pRSV-REV^51^ (a gift from Didier Trono, Addgene plasmid 12253) and 10 μg pMDLg/pRRE^51^ (a gift from Didier Trono, Addgene plasmid 12251) using Lipofectamine 2000 (Life Technologies 11668019) according to the manufacturer's protocol. Transfection medium was aspirated 8 h later and 6 ml fresh medium added. Forty-eight hours later, medium was harvested by centrifugation, filtered through a sterile 0.22 μm, 30 mm PES filter (Fisher Scientific 50-202-062), mixed 1:1 with HUVEC growth media (EBM-2+EGM-2 BulletKit, Lonza CC-3156 and CC-3162, respectively) and added to 80% confluent p2 HUVEC. The next day, medium was aspirated and fresh medium added. After 72 h, infected HUVEC were selected using 1 ng ml^−1^ puromycin (Sigma-Aldrich P9620-10 ml) in HUVEC growth medium, changed daily, for 2 weeks. Surviving HUVEC were then trypsinized, re-plated under 0.5 ng ml^−1^ puromycin selection and expanded.

### siRNA transfections

siRNAs used in this study are listed in Supplementary Table 2. HUVEC were plated at 20,000 cm^−2^ onto 6 cm dishes. The following day, ∼80% confluent HUVEC were transfected with 10 nM siRNA using Lipofectamine RNAiMax (Life Technologies 13778150) according to the manufacturer's protocol. The following day, HUVEC were trypsinized, coated onto Cytodex beads for 3D experiments or plated onto 12 mm coverslips for two-dimensional (2D) experiments and re-transfected with siRNA to ensure maximal knockdown persisted throughout the duration of the experiments. Residual cells were analysed by western blotting, to verify knockdown efficacy.

### Western blotting

HUVEC whole-cell lysates were prepared in RIPA buffer supplemented with protease/phosphatase inhibitor cocktail (Cell Signaling 5872S). Ten micrograms of whole-cell lysates were separated on 10% TGX Stain-Free FastCast SDS–PAGE gels (Bio-Rad 161–0183), followed by 2 min ultraviolet activation of TGX stain for total protein quantification. Proteins were transferred to polyvinylidene difluoride membranes (Bio-Rad 162–0177), blots were imaged under ultraviolet for total protein, blocked for 2 h in 5% non-fat milk (NFM) in PBS+0.1% Tween20 (PBST), then incubated overnight at 4 °C with primary antibody in 1% NFM in PBST (see Supplementary Table 1 for primary and secondary antibodies and concentrations). Horseradish peroxidase-conjugated secondary antibodies (Life Technologies goat anti-rabbit G21234 or rabbit anti-mouse 816720) were added for 2 h at RT in 1% NFM in PBST. Clarity ECL (Bio-Rad 170–5060) was used for detection. Films were digitized using an AlphaImager (ProteinSimple) gel scanning station and band densities calculated in FIJI^52^, following established guidelines^53^.

### Quantitative RT–PCR

Primers used in this study are listed in Supplementary Table 3. mRNA was collected from experimental samples using Trizol reagent (Life Technologies 15596026). cDNA was generated from 1 μg mRNA using iScript reverse transcription kit (Bio-Rad 170–8891) and diluted 1:3 in water. qRT–PCR was performed using iTaq Universal SYBR Green SuperMix (Bio-Rad 172–5121) on an ABI 7900HT Fast Real-Time PCR System (Life Technologies 4329001). Threefold serial dilutions of pooled cDNA were used to generate standard curves for each amplicon and data were analysed via the Pfaffl method^54^.

RNA was isolated from FACS-enriched zebrafish cells using RNA-easy Micro Plus kit (Qiagen) and reverse transcribed with Invitrogen's Superscript III First-Strand Synthesis Supermix (Invitrogen) according to the manufacturer's instructions. For qRT–PCR, NCBI's Primer-BLAST was used to design exon-spanning, gene-specific SybrGreen primers (see Supplementary Table 3). All primers were validated by high-resolution melt analysis, size confirmation and no-template controls. SybrGreen real-time PCR was performed in triplicate on the Viia7 real-time PCR system (Invitrogen). For quantification, the ΔΔCT method was used where raw CT values were normalized to *elongation factor alpha* (*eef1a1l1*) and paired sorting control, then calculated fold change as 2^(−ΔΔCT)^. Statistical significance was determined by one-sample *T*-test comparing with a reference value of onefold change.

### Zebrafish

Zebrafish (*Danio rerio*) embryos were maintained as previously described^9^. The following transgenic lines were used: *Tg(kdrl:GFP)*^*s843*^ (ref. 55), *Tg(kdrl:mCherry)* (gift of D. Stainier), *Tg(hsp70l:bmp2b)*^*fr13*^ (ref. 56), *Tg(Tp1bglob:eGFP)*^*um14*^ (ref. 57), *Tg(UAS:myc-Notch1a-intra)*^*kca3*^ and *Tg(hsp70l:Gal4)*^*1.5kca4*^ (ref. 58), and *Tg(fli1ep:dCas9-EnR;cmlc2:GFP)* (this study).

For all microinjections, one-cell-stage embryos were dechorionated using 20 mg ml^−1^ (W/V in ddH_2_O) pronase (Sigma P6911), then rinsed repeatedly using a total of 1 l H Buffer (60 mM NaCl, 2.3 mM NaHCO_3_, 1.1 mM CaCl_2_ and 0.67 mM KCl).

For Notch inhibition, embryos were treated with DAPT at 100 μM, sonicated in 1% dimethyl sulfoxide/1 × 1-phenyl 2-thiourea (PTU)/E3, or in 1% dimethyl sulfoxide/1 × 1-phenyl 2-thiourea (PTU)/E3 control, from 10 hpf until harvest. For Notch or BMP activation, embryos were heat-shocked at 26 hpf for 30 min at 40 °C or kept as unheat-shocked controls. For transgenesis, capped mRNA was generated from pCS2 FA Transposase^49^ using the T7 mMessage mMachine *in vitro* synthesis kit (Life Technologies AM1344). Five picograms each of transposase mRNA and Tol2 vector were injected into the cell of dechorionated one-cell-stage zebrafish embryos from *AB* (*fli1ep:dCas9-EnR;cmlc2:GFP*) or *Tg(kdrl:mCherry* (*fli1ep:smad6b-GFP;cmlc2:GFP)*, or *fli1ep:GFP;cmlc2:GFP*) in-crosses. Embryos were screened for cardiac GFP expression (transgenesis) and raised until harvest as F0 mosaics (*fli1ep:smad6b-GFP* or *fli1ep:GFP*) or raised to adulthood and screened for germline transmission (*fli1ep:dCas9-EnR;cmlc2:GFP*).

For global CRISPRi, WT embryos from AB parents were injected at the one-cell stage with 450 pg dCas9-EnR mRNA alone or in conjunction with 200 pg gene-targeting sgRNA. Embryos were analysed at 24 hpf for gross dorsal–ventral patterning defects consistent with early BMP manipulations^38^. For CRISPRi, >50 embryos from *Tg(hsp:bmp2b);Tg(kdrl:GFP) × Tg(fli1ep:dCas9-EnR;cmlc2:GFP)* crosses were injected using a PicoSpritzer III positive pressure micro-injection apparatus at the one-cell-stage with 200 pg (in 1 nl) of scrambled (scram) or *smad6b*-targeting sgRNAs. Embryos were raised at 28 °C in 1 × E3 and heat-shocked to overexpress Bmp2b at 26 hpf as described above. Embryos were fixed and imaged on an FV1200 confocal microscope at 44 hpf. sgRNA sequences used are listed in Supplementary Table 3.

For all zebrafish experiments, embryos were collected and sorted into treatments blindly with respect to genotype, to ensure appropriate randomization. For all experiments, any embryos that failed to gastrulate or exhibited severe developmental patterning defects were excluded from analysis. All animals are maintained in an institutional animal care and use committee (IACUC)-approved satellite facility according to public health service (PHS) policy on Humane Care and Use of Laboratory Animals.

### FACS sorting

Staged fish embryos were obtained by breeding hemizygous *Tg(Tp1:EGFP);Tg(myl7:dsRed)* adult zebrafish with homozygous *Tg(fli1:tdTomato;cmlc2:GFP)* fish by group spawning methods. Non-transgenic embryos (for use in setting FACS gates) were obtained by inbreeding WT (Tubingen) fish. Embryos from individual spawning groups were pooled into single clutch biological replicates. At 26 hpf, embryos were manually sorted for Tp1:EGFP expression. At 28–32 hpf, embryos were prepared for FACS sorting as previously described^19^. Briefly, embryos were manually dechorinated, yolk removed, then dissociated into single-cell suspension using a combination of TrypLE (Gibco) and FACSMax (Gentlantis). Cells were re-suspended in ice-cold L-15 (Gibco) supplemented with 5% heat-inactivated FBS (Hyclone), passed through 40 and 35 μm filters, then counterstained with Live/Dead fixable near-IR stain (Molecular Probes). A SH800 cell sorter (Sony) was used to sort live, tdTomato+ cells by Tp1:EGFP expression level and harvested directly into RTL lysis buffer, then stored at −20 for later RNA purification.

### ChIP assay

HUVEC were plated on 100 mm culture dishes and grown for 24 h and native protein–DNA complexes were cross-linked by treatment with 1% formaldehyde for 15 min. Simple ChIP Plus Enzymatic Chromatin IP kit (Cell Signaling 9005) was used per the manufacturer's protocol. Briefly, equal aliquots of isolated chromatin were subjected to immunoprecipitation with anti-RBPJ antibodies (Cell Signaling 5313s, Abcam ab25949) or rabbit IgG control at 2 μg per 500 μl. PCR reactions of immunoprecipitated DNA were performed to validate RBPJ binding on the Smad6 promoter. PCR primers used:

2: FWD: 5′-ATTAGCCGGGCATAGTGGTGCAT-3′

REV: 5′-TGCACTCAAGTGATTCTCGTGCCT-3′

3: FWD: 5′-AGGCGGATCACTTCAGGCAGGTCA-3′

REV: 5′-GCTCTGCACTCAAGTGATTCTCGT-3′.

PCR products were separated by gel electrophoresis and visualized by SYBRsafe (Invitrogen).

### Imaging and quantification

All images were acquired on an Olympus FluoView FV1200 confocal microscope, equipped with Green, Red and Far-Red lasers and detectors. Olympus OIB file formats were imported into FIJI using Bio-Formats Importer^20^ for analysis and quantification, as described below.

Branch frequency was measured by skeletonizing ES-cell-derived vessels or HUVEC-derived 3D sprouting angiogenesis assay vessels and calculating branches per mm using Image J (NIH). For branching parameters, the sprout length was measured from the base of the spout to the most distal end. The maximum branch angle was calculated at sprout junctions, excluding branches that fused with other sprouts distal to the junction. The percentage of tip cells was quantified in all multi-nucleated sprouts. Tip cells were defined as the cell nucleus located most distal in a sprout. In zebrafish, the percentage of somites containing angiogenic sprouts was calculated. The first 12 segments from the end of yolk extension were analysed as described^9^.

pSMAD1/5 fluorescence intensities were determined on a single-cell basis in 2D using FIJI as follows: HUVEC were stained for pSMAD1/5 (also FLAG if relevant) and incubated with DRAQ7 as described in detail above. The brightest slice in the DRAQ7 (nuclear) channel from confocal *z*-stack images was threshold adjusted into a binary image (black nuclei, white everywhere else). Next, using the ‘Analysis' → ‘Set Measurements' menu, analysis was re-directed from the binary nuclear image to the pSMAD1/5 channel and ‘Analyze' → ‘Analyze Particles' function was used with area cutoffs of 50–500 μm^2^ to return mean grey values per nucleus.

pSMAD1/5 fluorescence intensities were determined on a single-cell basis in 3D using FIJI as follows: pSMAD1/5 and DRAQ7 (nuclear) channels were independently compressed along the *z* axis by summing slices. DRAQ7 channel was threshold adjusted as described above and binary image used to re-direct measurements to the pSMAD1/5 channel as described above. The geodesic distance from the tip nucleus was measured along the length of the vessel from the most distal portion of the tip nucleus. For tip-cell distance:pSMAD1/5 fluorescence correlations, both distance and fluorescence were determined relative to the tip cell (0 μm distance and 100% fluorescence).

SMAD6 fluorescence intensities were determined on a single-cell basis in 2D as follows: HUVEC were stained for SMAD6 and FLAG, and incubated with Phalloidin to visualize cell boundaries as described in detail above. SMAD6 fluorescence intensity was measured by finding the brightest slice in the phalloidin channel, outlining individual cells and calculating integrated density within the SMAD6 channel with subtraction of background.

### Statistics

All statistical analyses were performed using Prism v6.05 (www.graphpad.com), with an *α* of 0.05. Data were tested for normal distribution using the D'Agostino and Pearson omnibus normality test within Prism. For two-sample data sets with equal variances (control -v- a single experimental condition) unpaired, two-tailed Student's *t*-test was used as reported in figure legends. For data sets with greater than two conditions and equal variances, one-way analysis of variance with Tukey's *post-hoc* test was used as reported in figure legends. For data sets with greater than two conditions and unequal variances, Kruskal–Wallis with Dunn's *post-hoc* test was used as reported in figure legends. No a priori sample-size power analyses were performed.

### Data availability

Data supporting the findings of this work are available within the article and its Supplementary Information files, and from the corresponding author on reasonable request.

## Additional information

**How to cite this article:** Mouillesseaux, K. P. *et al.* Notch regulates BMP responsiveness and lateral branching in vessel networks via SMAD6. *Nat. Commun.* **7,** 13247 doi: 10.1038/ncomms13247 (2016).

**Publisher's note:** Springer Nature remains neutral with regard to jurisdictional claims in published maps and institutional affiliations.

## Supplementary Material

Supplementary Information Supplementary Figures 1-5 and Supplementary Tables 1-3.
