# Supplementary material for: Notch regulates BMP responsiveness and lateral branching in vessel networks via SMAD6
Source: Nat Commun. 2016 Nov 11;7:13247. doi: 10.1038/ncomms13247 (PMC5114582; doi:10.1038/ncomms13247)
Supplement: Supplementary Information — Supplementary Figures 1-5 and Supplementary Tables 1-3. [file ncomms13247-s1.pdf]

SUPPLEMENTARY INFORMATION: Supplementary Figures, Figure Legends and Tables.

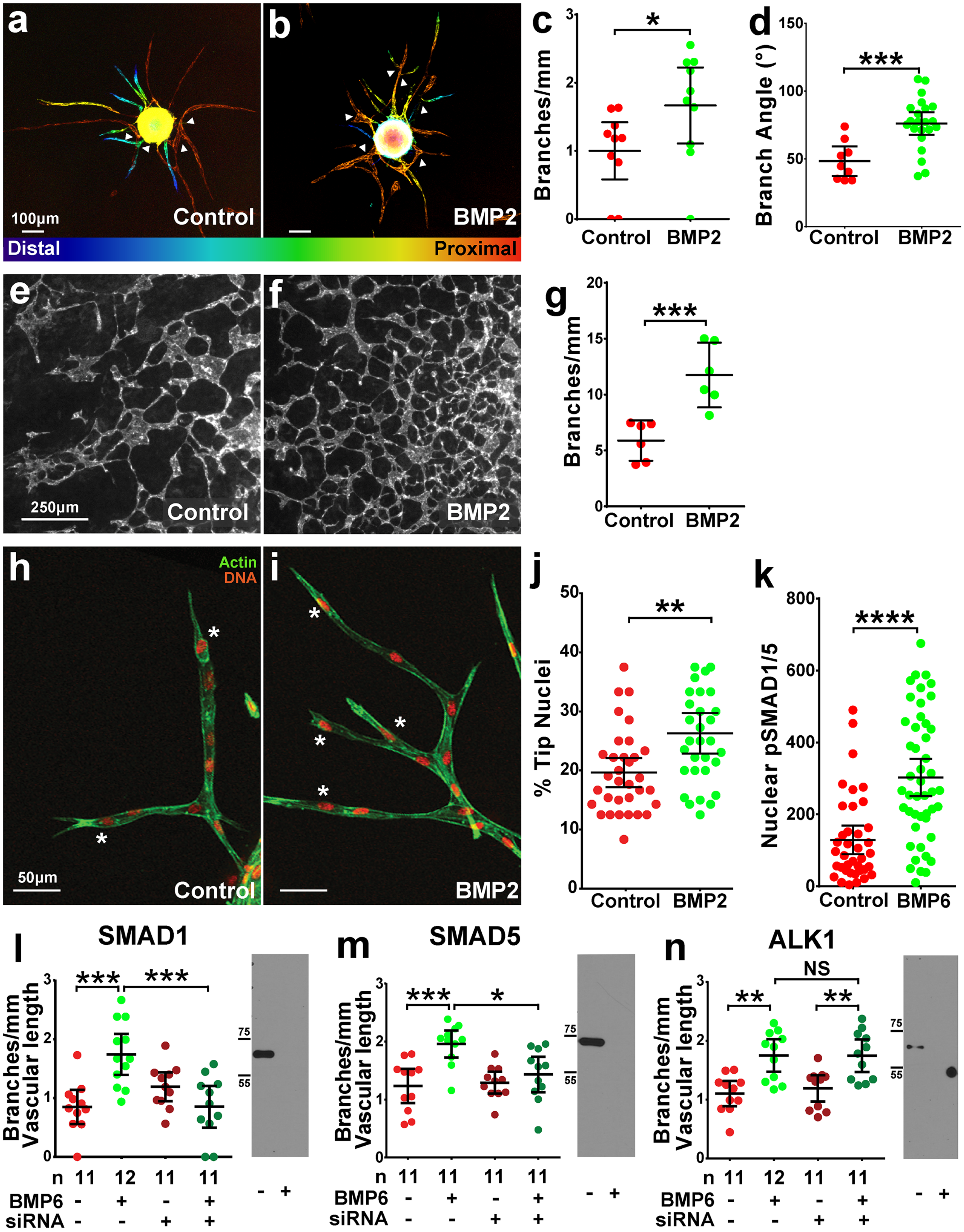

Supplementary Figure 1 | Pro-angiogenic BMP ligands promote lateral branching.

(a-b) HUVEC 3D sprouting assay +/- BMP2, visualized with phalloidin (actin) and depth-encoded. Arrowheads indicate branches. **(c-d)** Quantification of (c) branches/mm (n = 10 control and 10 BMP2 beads) and (d) branch angle (n = 9 control and 22 BMP2 angles**)**, representative of 3 independent experiments. Error bars, mean +/- 95% CI. *, p ≤ 0.05; ***, p ≤ 0.001 by Student's t-test. **(e-f)** Mouse ES cell-derived vessels stained for PECAM-1 with indicated treatments. **(g)** Quantification of branches/mm, representative of 3 independent experiments (n = 6 biological replicates for each condition). Error bars, mean +/- 95% CI. ***, p ≤ 0.001 by unpaired, two-tailed Student's t-test. **(h-i)** HUVEC 3D sprouting assay with control **(h)** or BMP2 **(i)** visualized with phalloidin (actin) or DRAQ7 (DNA). Asterisks, tip cell nuclei. **(j)** Quantification of tip nuclei/total nuclei/field, representative of 3 independent experiments (n = 33 control and 31 BMP2 sprouts). Error bars, mean +/- 95% CI. **, p ≤ 0.01 by unpaired, two-tailed Student's t-test. **(k)** Quantification of mean pSMAD1/5 intensity per nucleus from Fig. 1h-i, representative of 2 independent experiments**.** Error bars, mean +/- 95% CI. ****, p ≤ 0.0001 by unpaired, two-tailed Student's t-test. **(l-n)** HUVEC 3D sprouting assay with control or BMP6 and indicated siRNA treatments, representative of 2 independent experiments (n indicated on graphs). Error bars, mean +/- 95% CI. *, p ≤ 0.05; **, p ≤ 0.01; ***, p ≤ 0.001 by 1-way ANOVA, with Tukey's post-hoc. Right panels, Western Blot of relevant proteins to show siRNA knockdown (**Supplementary Fig. 5b**). Left of panels, 55 kDa and 75 kDa markers.

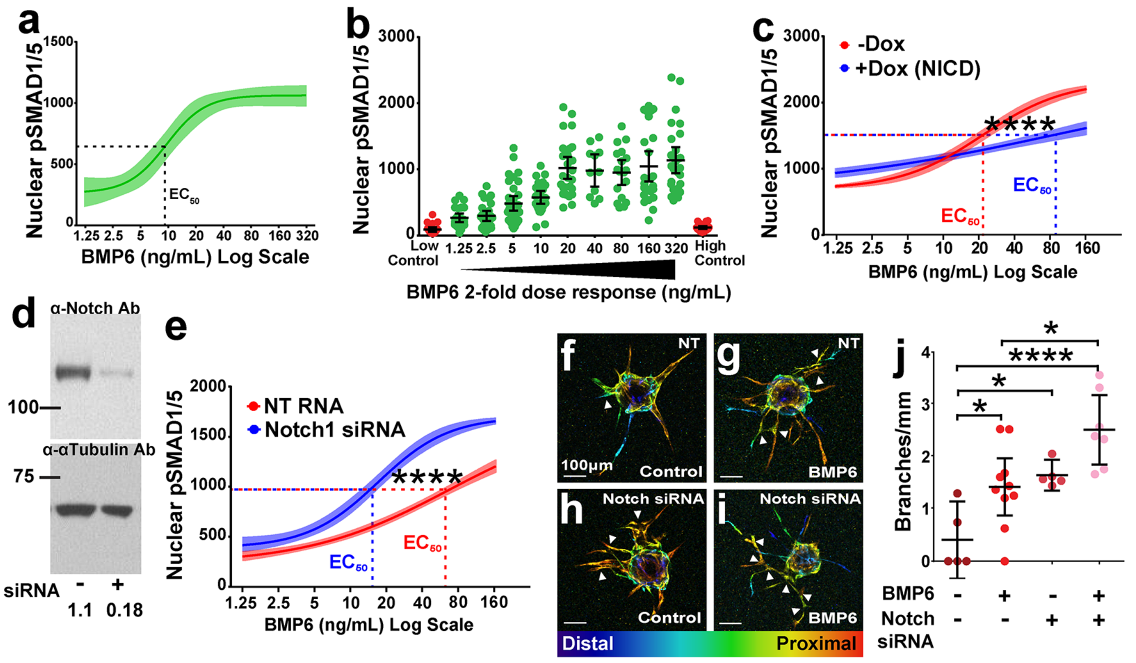

Supplementary Figure 2 | Notch affects HUVEC BMP-responsiveness.

(a) pSMAD1/5 nuclear fluorescence dose-response curve of HUVEC to increasing BMP6 for 90 min as indicated on the X-axis, representative of 2 independent experiments. Data are 4-parameter best-fit curves (solid lines) +/- 95% confidence bands (filled areas). **(b)** Raw spread-scatter data for pSMAD1/5 nuclear fluorescence response curve of HUVEC to increasing 90 min BMP6 treatments shown in panel a, representative of 2 independent experiments. Low and high controls, solvent % matched to the lowest/highest ligand concentrations. Data points, mean nuclear pSMAD1/5 fluorescence of individual nuclei; bars, population means +/- 95% CI. **(c)** BMP6 2-fold dose-response curves (indicated on X-axis) in HUVEC after 24 hr Notch activation (+DOX, blue line) compared to control (-DOX, red line). Data are 4-parameter best-fit curves (solid lines) of the population means +/- 95% confidence bands (shaded bands), representative of a single experiment. ****, $p \leq 0.0001$ by non-linear regression. **(d)** Western blot 48 hr post-transfection of indicated siRNAs, showing knockdown of Notch1 in HUVEC. Left, size markers (kDa). Below, ratio of Notch/α-tubulin signal (**Supplementary Fig. 5c**). **(e)** BMP6 2-fold dose-response curves (indicated on X-axis) in HUVEC 48 hr post-transfection with NT (red line) or Notch1-targeting siRNAs (blue line). Data are 4-parameter best-fit curves (solid lines) of the population means +/- 95% confidence bands (shaded areas), representative of 2 independent experiments. ****, $p \leq 0.0001$ by non-linear regression. **(f-i)** 3D HUVEC sprouting assay with control (f-g) or Notch siRNAs (h-i) and with control (f,h) or BMP6 (g,i) daily from 2d-7d post-embedding, visualized with phalloidin (actin) or DRAQ7 (DNA) and depth-encoded. **(j)** Quantification of branches/mm per bead from a single experiment (n = 5 NT/Control, 10 NT/BMP6, 5 Notch siRNA/Control, and 7 Notch siRNA/BMP6 beads). Data points, beads; bars, means +/- 95% CI. *, $p \leq 0.05$; ****, $p \leq 0.0001$ by 1-way ANOVA with Tukey's post-hoc.

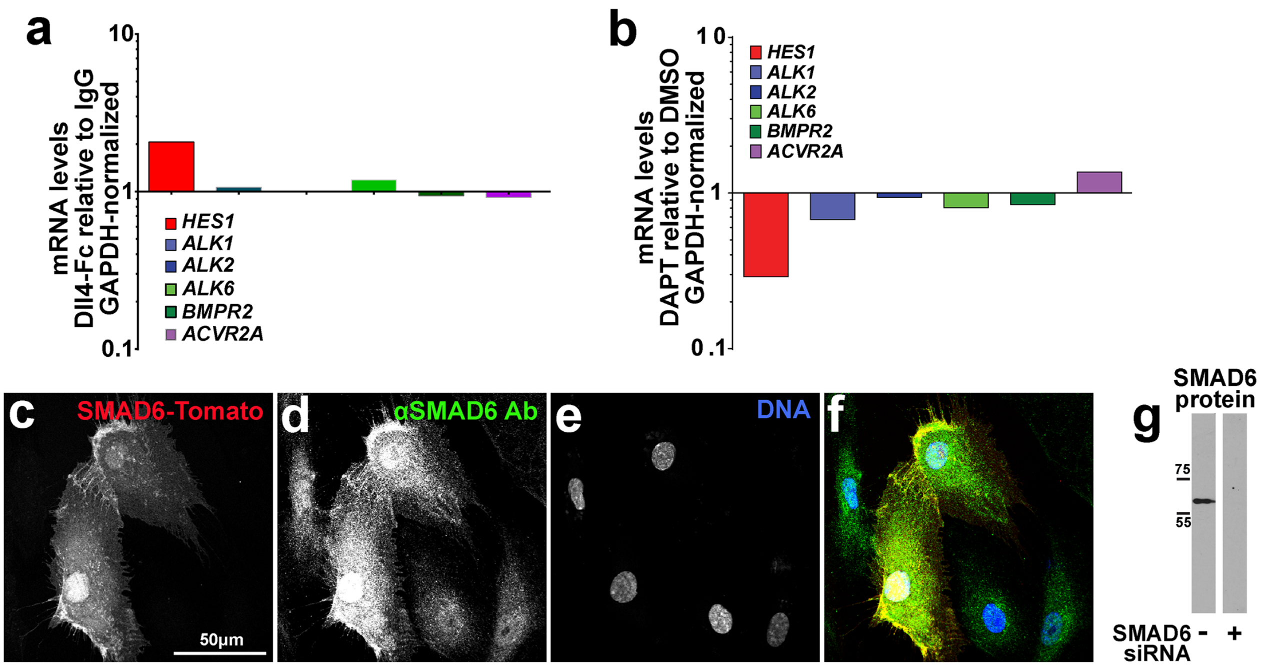

Supplementary Figure 3 | Lack of Notch regulation of BMP receptors and validation of SMAD6 over-expression and knockdown reagents.

(a-b) qRT-PCR for BMP pathway receptor mRNAs and the Notch target, *HES1*, with Notch gain-of-function (Dll4-Fc, (a)) or loss-of-function (DAPT, (b)). Data from single of 3 experiments, displayed as a log-conversion of the $\Delta\Delta C_T$ vs. controls. **(c-f)** HUVEC infected with inducible SMAD6-tdTomato, 16 hr post-DOX treatment and visualized for SMAD6-tdTomato (red), antibody-detected SMAD6 (green), or DNA (blue). **(g)** Western blot of HUVEC transfected with indicated siRNAs. 55 and 75kDa molecular weight markers shown. (**Supplementary Fig. 5d**).

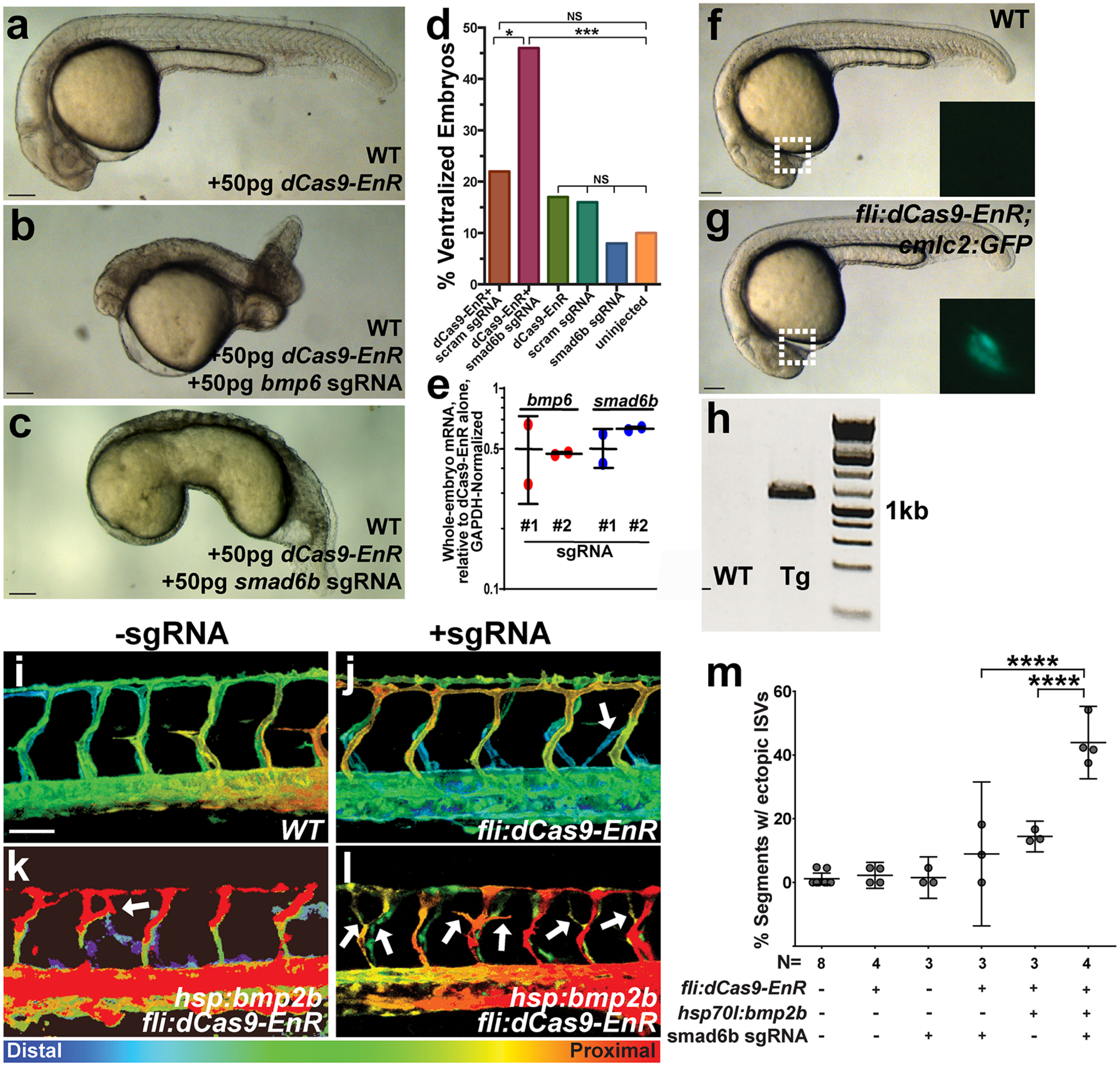
a
WT
+50pg dCas9-EnR
b
WT
+50pg dCas9-EnR
+50pg bmp6 sgRNA
c
WT
+50pg dCas9-EnR
+50pg smad6b sgRNA
d
% Ventralized Embryos
NS
*
***
NS
dCas9-EnR+ scram sgRNA
dCas9-EnR+ smad6b sgRNA
dCas9-EnR
scram sgRNA
smad6b sgRNA
uninjected
e
Whole-embryo mRNA, relative to dCas9-EnR alone, GAPDH-Normalized
bmp6
smad6b
#1 #2 #1 #2
sgRNA
f
WT
g
fli:dCas9-EnR; cmlc2:GFP
h
1kb
WT Tg
-sgRNA
+sgRNA
i
WT
j
fli:dCas9-EnR
k
hsp:bmp2b
fli:dCas9-EnR
l
hsp:bmp2b
fli:dCas9-EnR
Distal
Proximal
m
% Segments w/ ectopic ISVs
****
****
N= 8 4 3 3 3 4
fli:dCas9-EnR - + - + + +
hsp70l:bmp2b - - - - + +
smad6b sgRNA - - + + - +

Supplementary Figure 4 | SMAD6 mediates Notch-dependent suppression of BMP signaling and angiogenesis.

(a-c) CRISPRi phenotypes with sgRNA manipulations (Scale bar, 100 μm). **(a)** WT control injected with 50pg dCas9-EnR mRNA, **(b)** dCas9-EnR + 50pg *bmp6* sgRNA (predicted loss-of-function for BMP signaling), **(c)** dCas9-EnR + 50pg *smad6b* sgRNA (predicted gain-of-function for BMP signaling); two different sgRNAs produced the ventralized phenotype. **(d)** Quantification of early embryonic phenotypes of indicated injections. (#embryos: dCAS+scram=36; dCAS+smad6=57; dCas=48; scram=55; smad6=76; uninjected=101). X^2 analysis; *, p≤0.05; ***, p≤0.001; NS, not significant. **(e)** qRT-PCR of whole embryos for indicated mRNAs targeted with two unique sgRNAs each. Data points, replicated experiments using 25 embryos per condition, displayed as a log-conversion of the $\Delta\Delta C_T$ vs. controls. **(f-g)** WT (f) or dCas9-EnR+ (with a linked *myl7:GFP* transgene - inset) (g) F1 embryos from *Tg(fli:dCas9-EnR)* and *Tg(hsp70l:bmp2b)* crosses were indistinguishable by gross morphology and developmental staging. Scale bar, 100 μm. **(h)** RT-PCR with dCas9-EnR primers from 24hpf mRNA from WT (left) or *Tg(fli:dCas9-EnR)* (Tg, right) embryos. dCas9-EnR PCR product is at expected size (~1.2kb) (**Supplementary Fig. 5e**). All embryos are at 24 hpf. **(i-l)** Heat-shocked F1 embryos (heat shock at 26 hpf, analyze at 44-46 hpf) from *Tg(fli:dCas9-EnR)* and *Tg(hsp70l:bmp2b;Tg(kdrl:GFP)* crosses, uninjected or injected with *smad6b* sgRNAs. Arrows, ectopic ISV sprouts. Panels k and l have Z planes with ectopic venous sprouts removed. **(m)** Quantification of arterial vascular defects (% segments with ectopic ISVs) in heat-shocked embryos of indicated genotypes, representative of 2 independent experiments, N values on graph. Error bars, mean +/- SEM; *, p≤0.05; ****, p ≤ 0.0001 by 1-way ANOVA, with Tukey's post-hoc.

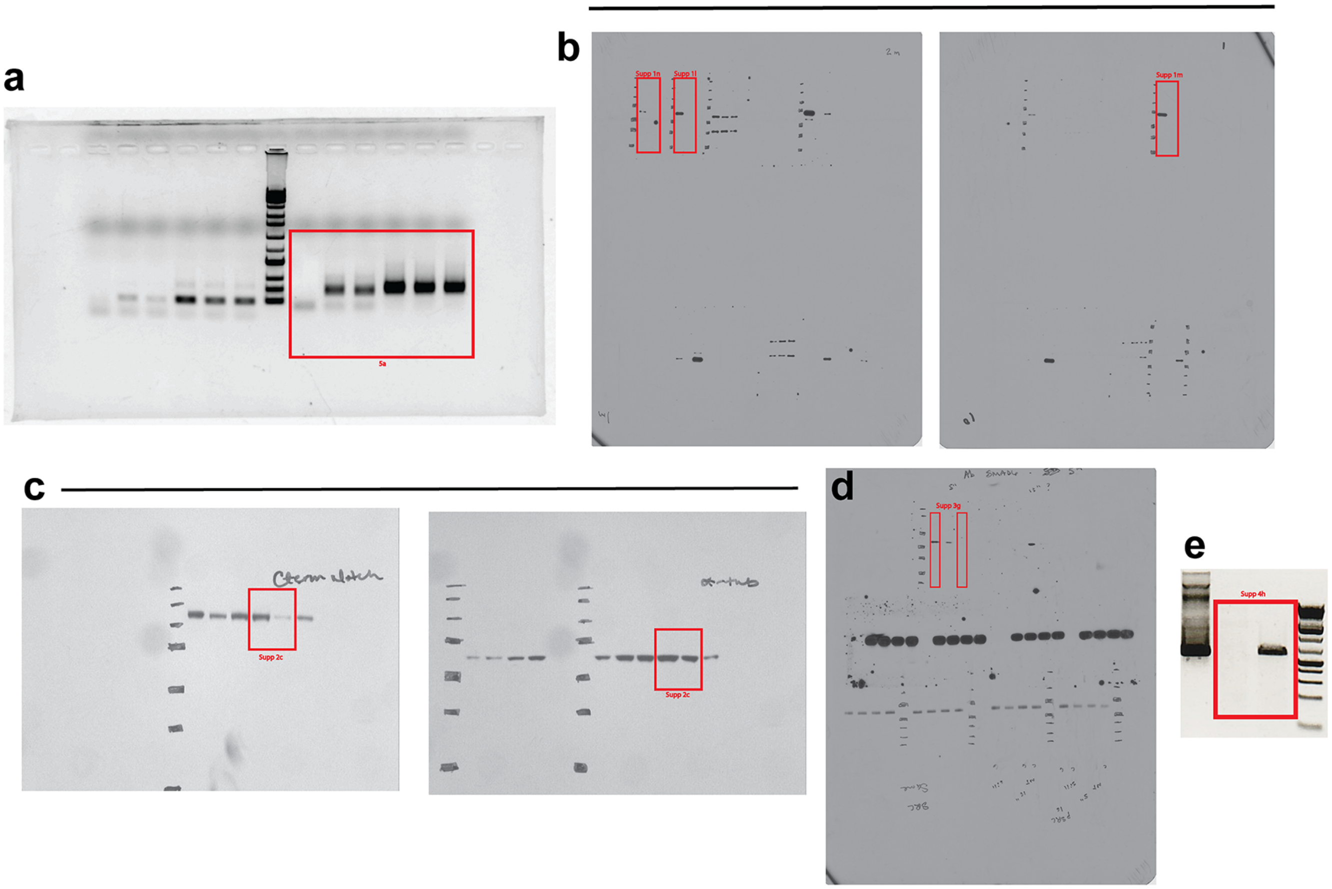

Supplementary Figure 5 | Original gels and Western blots.

(a) Gel Fig. 5a. **(b)** Blots Supplementary Fig. 1. **(c)** Blots Supplementary Fig. 2. **(d)** Blot Supplementary Fig. 3. **(e)** Gel Supplementary Fig. 4.

Supplementary Table 1. Antibodies.

Antibody	Species	Assay	Dilution	2° Ab Dilution	1° Ab Incubation	Blocking Buffer	Ab Buffer	Vendor	Catalog #	Observed MW
α-Alk1	RabMAb	WB	1:2000	1:2000	O/N @ 4°	5% NFM/PBST	1% NFM/PBST	AbCam	ab108207	65kDa
a-FLAG M2 Monoclonal	MusMAb	WB	1:2000	1:5000	O/N @ 4°	5% NFM/PBST	1% NFM/PBST	Sigma	F1804	
α-pSmad1(S463(5))/5(S463(5))	RabMAb	WB	1:5000	1:5000	O/N @ 4°	5% NFM/PBST	1% NFM/PBST	Cell Signaling	9516	60kDa
α-SMAD1	RabMAb	WB	1:20000	1:20000	O/N @ 4°	5% NFM/PBST	1% NFM/PBST	Cell Signaling	6944	60kDa
α-SMAD5	RabMAb	WB	1:5000	1:5000	O/N @ 4°	5% NFM/PBST	1% NFM/PBST	Cell Signaling	12534	
α-SMAD6	RabPAb	WB	1:5,000	1:10000	O/N @ 4°	5% NFM/PBST	1% NFM/PBST	AbCam	ab13727	50kDa

Antibody	Species	Assay	Dilution	2° Ab Dilution	1° Ab Incubation	Blocking Buffer	Ab Buffer	Vendor	Catalog #	Observed MW
α-pSmad1(S463(5))/5(S463(5))	RabMAb	2D IF	1:1000	1:1000	O/N @ 4°	5% GS/1% BSA/0.3% Triton-X100/PBS	5% GS/1% BSA/0.3% Triton-X100/PBS	Cell Signaling	9516	
α-pSmad1(S463(5))/5(S463(5))	RabMAb	3D IF	1:250	1:1000	48h @ 4°	5% GS/1% BSA/0.3% Triton-X100/PBS	5% GS/1% BSA/0.3% Triton-X100/PBS	Cell Signaling	9516	
α-VE-cadherin	RabMAb	2D IF	1:2000	1:2000	O/N @ 4°	5% GS/1% BSA/0.3% Triton-X100/PBS	5% GS/1% BSA/0.3% Triton-X100/PBS	Cell Signaling	2500P	

Antibody	Species	Assay	Vendor	Catalog #
α-RBPJ	RabMab	ChIP	Cell Signaling	5313s
α-RBPJ	RabPab	ChIP	Abcam	ab25949

Supplementary Table 2. siRNA Oligonucleotides.

siRNA target	Catalog Number	Targeted Exon	RefSeq	Vendor
Non-targeting control	4390847	N/A	N/A	Life Technologies
SMAD6	4427037 (s8410)	4	NM_005585	Life Technologies
SMAD6	4427037 (s8411)	3	NM_005585	Life Technologies
Notch1	4427037 (s9633)	22,23	NM_017617	Life Technologies
Notch1	4427037 (s9635)	27	NM_017617	Life Technologies
SMAD1	4427037 (s8395)	3	NM_005900	Life Technologies
SMAD1	4427037 (s8396)	7	NM_005900	Life Technologies
SMAD5	4427037 (s8406)	6	NM_001001419	Life Technologies
SMAD5	4427037 (s8407)	4	NM_001001419	Life Technologies
ACVRL1 (ALK1)	4427037 (s986)	2	NM_001077401	Life Technologies
ACVRL1 (ALK1)	4427037 (s987)	9	NM_001077401	Life Technologies

Supplementary Table 3. RT-PCR Primers and sgRNA Sequences.

Gene (Homo sapiens)	F Primer (5'-->3')	R Primer (5'-->3')	Vendor	Catalog Number
ACVRL1 (ALK1)	Proprietary sequence	Proprietary sequence	BioRad	qHsaCID0021337
ACVR1 (ALK2)	Proprietary sequence	Proprietary sequence	BioRad	qHsaCID0012283
ACVR2A	Proprietary sequence	Proprietary sequence	BioRad	qHsaCID0014728
BMPR1B (ALK6)	Proprietary sequence	Proprietary sequence	BioRad	qHsaCID0021330
BMPR2	Proprietary sequence	Proprietary sequence	BioRad	qHsaCID0008240
GAPDH	Proprietary sequence	Proprietary sequence	BioRad	qHsaCID0038674
HES1	ATCTGAGCACAGAAAGTCATCAAAG	GGATGCTCTGAAGAAAGATAGCTC	N/A	N/A
SMAD6	Proprietary sequence	Proprietary sequence	BioRad	qHsaCID0017103

Gene (Danio rerio)	F Primer (5'-->3')	R Primer (5'-->3')	Vendor	Catalog Number
bmp6	ATGTGAGTTTCAGAGAATTGAGCTG	ACCAGTGTCTGTACTATTGCATGAT	N/A	N/A
gapdh	TGTGATGGGAGTCAACCAGGACAA	TTAGCCAGAGGAGCCAAGCAGTTA	N/A	N/A
smad6b	GTCAGATTCCACACTGTCTTACACT	ACGTTACACCAGTGACTCTGCTT	N/A	N/A

FACS sorted EC primers				
ef1a	AGGACATCCGTCGTGGTAAT	AGAGATCTGACCAGGGTGGTT	Eurofins	N/A
gapdh	TGACCTGATGGCACACATGG	TGGGAGAATGGTCGCGTATC	Eurofins	N/A
smad6b	AGGGGAATTCTCAGATGCCAG	GGTACACCGTATAGAGGCGG	Eurofins	N/A
notch1b	TGCGAGAACAACACACCTGA	CTGGCAGTAGTTGCCAGTGA	Eurofins	N/A
dll4	AACTGCGAGAGAAGGATGGAC	CCCAGGATCAAACACAAGCCA	Eurofins	N/A
efnb2	ACCCTACCAGTTACCCTCCC	CCATCTCCCTTATCTTCCCCA	Eurofins	N/A
lyve1	ACATGCAGGTTTGGTTGGGT	CTGATCTCAGCTCTCCAAGTGA	Eurofins	N/A

sgRNA (Danio rerio)	Sequence		Vendor	Catalog Number
smad6b #1	AACCCTAAGGAAAGCCCT		N/A	N/A
smad6b #2	CGACCCGCTCGGGGTCAT		N/A	N/A
bmp6 #1	TTCTTCACTGGTTCCTAC		N/A	N/A
bmp6 #2	ACAGCGCGTCTGTCTCAG		N/A	N/A
scrambled (scram)	GCTGATCTATCGCGGTCGTC		N/A	N/A
